# ACCBN: ant-Colony-clustering-based bipartite network method for predicting long non-coding RNA–protein interactions

**DOI:** 10.1186/s12859-018-2586-3

**Published:** 2019-01-09

**Authors:** Rong Zhu, Guangshun Li, Jin-Xing Liu, Ling-Yun Dai, Ying Guo

**Affiliations:** 1 School of Information Science and Engineering, Central South University, Changsha, 410083 China; 2 School of Information Science and Engineering, Qufu Normal University, Rizhao, 276826 China

**Keywords:** LncRNA–protein interaction, Ant colony clustering, Bipartite network, Predicting

## Abstract

**Background:**

Long non-coding RNA (lncRNA) studies play an important role in the development, invasion, and metastasis of the tumor. The analysis and screening of the differential expression of lncRNAs in cancer and corresponding paracancerous tissues provides new clues for finding new cancer diagnostic indicators and improving the treatment. Predicting lncRNA–protein interactions is very important in the analysis of lncRNAs. This article proposes an Ant-Colony-Clustering-Based Bipartite Network (ACCBN) method and predicts lncRNA–protein interactions. The ACCBN method combines ant colony clustering and bipartite network inference to predict lncRNA–protein interactions.

**Results:**

A five-fold cross-validation method was used in the experimental test. The results show that the values of the evaluation indicators of ACCBN on the test set are significantly better after comparing the predictive ability of ACCBN with RWR, ProCF, LPIHN, and LPBNI method.

**Conclusions:**

With the continuous development of biology, besides the research on the cellular process, the research on the interaction function between proteins becomes a new key topic of biology. The studies on protein-protein interactions had important implications for bioinformatics, clinical medicine, and pharmacology. However, there are many kinds of proteins, and their functions of interactions are complicated. Moreover, the experimental methods require time to be confirmed because it is difficult to estimate. Therefore, a viable solution is to predict protein-protein interactions efficiently with computers. The ACCBN method has a good effect on the prediction of protein-protein interactions in terms of sensitivity, precision, accuracy, and F1-score.

## Background

LncRNA refers to a class of non-coding RNAs that are greater than 200 nucleotides in length and do not encode proteins [1, 2]. RNA In the human transcription, only about 1% of RNA encodes proteins, most of which belong to long non-coding RNAs [3]. In the past few years, more and more evidence shows that lncRNA is closely related to the biological behaviors such as tumor development, invasion, and metastasis. With the in-depth study of genomics, a good deal of studies has shown that lncRNA has an undoubted regulation effect on tumors. LncRNA is also involved in the formation of many diseases [4]. The diversity and complexity of lncRNA function is due to interaction with multiple proteins [5], which regulates multiple cellular processes by binding to proteins to achieve their specific functions.

In recent years, bioinformatics has developed rapidly, and a good deal of lncRNAs has also been found. Although some lncRNAs have been well studied, the function of most lncRNAs remains unknown and needs further study. Typically, most lncRNAs act by interacting with the corresponding RNA binding proteins [6]. As a result, a detection of lncRNA-protein interactions is very important for studying the function of lncRNA. In actual research, experimental identification of lncRNA-protein interactions is expensive. Therefore, it is crucial to develop effective computational prediction methods. In recent years, many scholars have developed many computational prediction methods [6–18].For example, Bellucci et al. introduced the catRAPID method [7] by thinking about the secondary structure, hydrogen bonds, and van der Waals forces between lncRNAs and proteins. Muppirala et al. proposed the RPISeq method [10] only by considering the sequence information of lncRNA and protein. Lu et al. introduced the lncPro method [11], which not only uses the secondary structure, hydrogen bonding, van der Waals force characteristics, but also uses the Fisher linear discriminant method to obtain prediction scores.

The aforementioned algorithms are based on the sequence features. However, in general, lncRNAs often exhibit low sequence conservation [19], and the effect of predicting interactions based on lncRNA-based sequence features is not ideal. With the development of bioinformatics technologies, lncRNA–protein interaction networks have enabled to construct, and biological network-based methods have been applied to the studies on protein prediction studies. MengquGe et al. introduced a lncRNA–protein bipartite network inference (LPBNI) [14] to predict lncRNA–protein interactions. LPBNI can effectively predict new lncRNA-protein pairs through the use of the lncRNA-protein bipartite network.

In this paper, we present a novel prediction method named ACCBN. The ACCBN method can predict unobserved lncRNA-protein interactions more effectively for the following reasons. Firstly, lncRNA is represented as a feature vector and lncRNA is used as a data point in the feature space. Secondly, the similarity is enhanced by using the Ant Colony Clustering method. Thirdly, an effective prediction of lncRNA-protein interactions is achieved by applying a lncRNA-protein bipartite network.

## Methods

### The basic principle of ant colony clustering

Clustering is the important content of data mining, which is an unsupervised learning process. The basic principle is to cluster data sets according to different features between data and find the hidden pattern in data. In recent years, the application of clustering algorithms has been a research hotspot. At present, clustering algorithms can be roughly divided into four categories, namely hierarchical, partitioning, density-based and grid-based clustering methods. Recently, scientists have proposed an ant colony clustering algorithm based on the intelligence of ant colony.

The first studies of ant-based clustering algorithms were performed by Deneubourg et al.. Deneubourg et al. proposed a basic model that allowed ants to randomly move, pick up, and deposit objects in clusters on the basis of the number of similar surrounding objects. The clustering method based on the food-seeking principle of ants has got the name from the food-seeking process, in which an ant releases a chemical substance called pheromone along the path and other ants can perceive this pheromone. The ant colony behavior done by a large number of ants is presented as a kind of positive feedback of information, and clustering is realized through this kind of positive feedback mechanism. In the clustering process based on the food-seeking principle of ants, the data to be clustered are regarded as ants of different properties and the clustering center is considered as the food source to be sought. Therefore, the data clustering process can be considered to be the process of ants seeking for the food source. During each search cycle, the ants would calculate the transition probability (which is concerned with the amount of information to reach the clustering center) and heuristic information to decide the next transition location.

The idea of ant colony clustering algorithm based on ant colony foraging principle is as follows:

First of all, the initialization of the algorithm, initialize the pheromone on various paths, set*T*_*ij*_(0) = 0, and set various parameter values, such as the radius*r* of cluster, *p*_0_ and α, β is conducted.

And then, during the algorithm operation process, the pheromone *T*_*ij*_(*t*) on various paths is calculated:

1 $$ {T}_{ij}(t)\left\{\begin{array}{c}1,{d}_{ij}\le r\\ {}0.{d}_{ij}>r.\end{array}\right. $$

During the algorithm operation process, the probability that the data object *x*_*i*_ and data object *x*_*j*_ belong to the same cluster is calculated:

2 $$ {\displaystyle \begin{array}{l}{p}_{ij}(t)=\frac{{\left[{T}_{ij}(t)\right]}^{\alpha }{\left[{\eta}_{ij}(t)\right]}^{\beta }}{\sum \limits_{j=1}^k{\left[{T}_{ij}(t)\right]}^{\alpha }{\left[{\eta}_{ij}(t)\right]}^{\beta }},\\ {}\end{array}} $$

where, if *p*_*ij*_(*t*) > P_0_, it indicates that data object *x*_*i*_and data object *x*_*j*_ belong to the same cluster, and combine *x*_*i*_ to the field of *x*_*j*_. *α*is the heuristic factor of information, which reflects the importance of accumulated pheromone *T*_*ij*_(*t*) by ants during the operation. *β*is the expected heuristic factor, which reflects the importance of heuristic information *η*_*ij*_(*t*) of ants during the movement. *T*_*ij*_(*t*)refers to the pheromone on the path from data *x*_*i*_ to the *j* − *th* clustering center in the *t* − *th* clustering. *η*_*ij*_(*t*)is the visibility function, which reflects a priori certainty factor of ants during the movement. ${\eta}_{ij}=\frac{1}{d_{ij}}$, *d*_*ij*_ refers to the Euclidean distance from the data object *i* to the clustering center *c*_*j*_, which is presented as follows:

3 $$ {d}_{ij}={\left(\sum \limits_{k=1}^m{\left|{x}_{ik}-{x}_{jk}\right|}^2\right)}^{\frac{1}{2}}\kern0.5em k\in \left\{1,2,\cdots, m\right\}. $$

However, every time ants complete one clustering, the clustering center will change, and the pheromones from each data to the clustering center are adjusted according to the following rule:

4 $$ {T}_{ij}\left(\mathrm{t}+1\right)=\left(1-\rho \right){T}_{ij}(t)+\varDelta {T}_{ij}(t), $$

5 $$ \varDelta {T}_{ij}(t)=\frac{Q}{d\left({x}_i,{c}_j\right)}, $$

where *ρ* refers to the volatilization degree of pheromone; (1 − *ρ*) refers to the residual degree of pheromone; *ΔT*_*ij*_(*t*) represents the increment of pheromone from data *i* to cluster *j* during this cycle; *Q* is a constant value. The bigger the *Q*value, the faster the pheromone accumulates on the path where the ants have passed. In a word, the *Q* value affects the convergence speed of the algorithm to a certain degree.

Each transition of ants between different clustering centers will result in a change of clustering center, and the next clustering process will start until the clustering result is stable. In this process, most initial parameters are determined by the experience, and the common ranges are*α* ∈ (0, 5), *β* ∈ (0, 5), *ρ* ∈ (0.1, 0.99), *Q* ∈ (1, 10000).

### Improved ant colony clustering algorithm

**Fig. 1:**
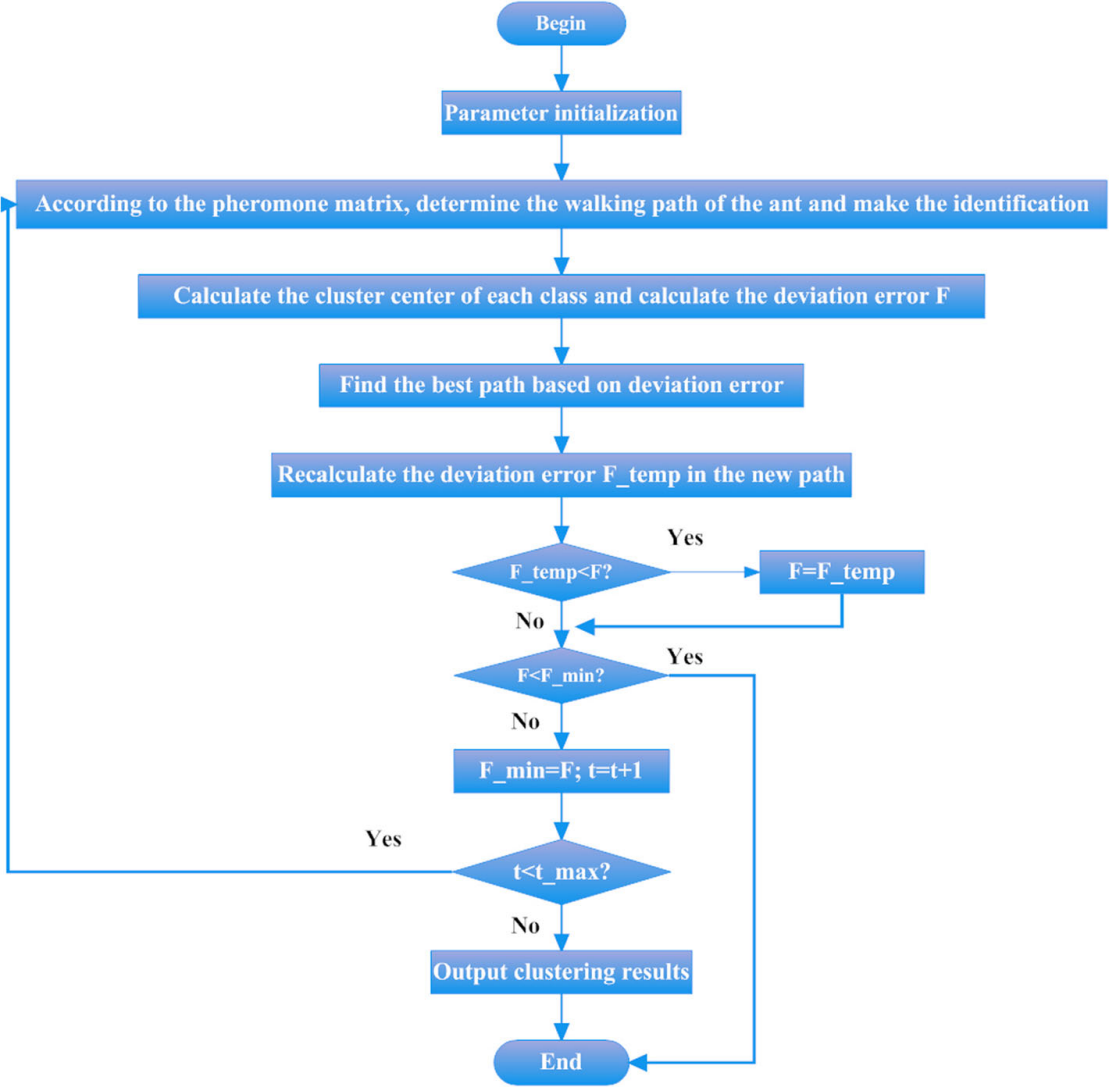
Flowchart of the ant colony clustering algorithm with mutation characteristics

During the application of ant colony clustering algorithm, the algorithm has slow convergence speed, and especially during the initial period of iteration, due to a slow update of pheromone, it is difficult to distinguish pheromones on each path. However, during the later period of iteration, the pheromones on some paths will be continuously accumulated, as a result, it would be more probable for ants to choose the path with more pheromones in a later operation, but it cannot ensure that the solution is a global optimal pollution, which results in the premature phenomenon. There has been a significant amount of research recently conducted on the improved performance and wider applications of ant colony clustering algorithms. The ant colony clustering algorithm with variation characteristics is thus proposed here for an improvement. The algorithm flow chart is as shown in Fig. 1.

As Fig. 1 shows, F is the mean square error of various properties of various sample points *F* to the clustering center; *F* _ *temp* refers to the mean square error of various properties of various sample points to their corresponding clustering center under the variation path; *F* _ min represents the minimum mean square error of various properties to their corresponding clustering center in *t* − *th* iterations.

The variation times in the algorithm are random. However, through the introduction of variation, the algorithm can break through its original operation mechanism, and in other words, during the tolerable convergence process is optimized at random, which improves the performance of the original algorithm in a certain degree.

### Constructing the lncRNA–protein bipartite network

**Fig. 2:**
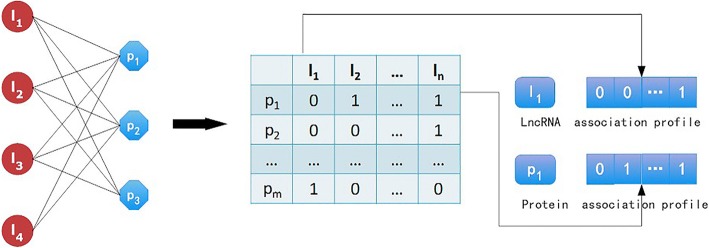
The lncRNA-protein association bipartite network

We use a graph *G*(*L*, P, E) to describe the lncRNA-protein interaction network. *L* = {*l*_1_, *l*_2_, ⋯, *l*_*n*_}denotes lncRNA set. P = {p_1_, *p*_2_, ⋯, *p*_*m*_}denotes the protein set. E = {e_*ij*_| *l*_*i*_ ∈ *L*, p_*j*_ ∈ *P*}denotes the edge set, and e_*ij*_ is the edge connecting the nodes *l*_*i*_ and p_*j*_. The bipartite network is shown in Fig. 2.

In this section, we study the association profile of lncRNA and the associated profile of proteins based on a binary network. In Fig. 2, lncRNA association profiles and protein association profiles are corresponding to row vectors and column vectors of the association matrix. Association profiles are the very significant information obtained from the lncRNA-protein association network. We use the association profiles to build models and predict lncRNA–protein interactions.

Referring to [20],we calculated the similarity of lncRNA-lncRNA and the similarity of protein-protein by exploiting linear neighborhood similarity (LNS). The prediction model is then built by using marker propagation.

For given *m* lncRNAs, a similarity matrix *W* is computed, and then we make up a directed graph in which lncRNA is used as the node and its similarity is used as the weight of the edges. We use the known association between the specified protein and all lncRNAs as the initial mark for the node. For the protein*P*_*k*_, the *k* − *th* column of the association matrix *Z*is known as the initial labels of the nodes, and is written as*Z*(:, *k*). In the directed graph, for the labels of nodes are updated, the labels of neighbors with the probability *ρ* are absorbed and reserve the initial labels with the probability 1 − *ρ*. Let ${P}_k^t$ represent the labels of nodes at *t* − *th* iteration, we use the following formula to infer the update of step *t* − 1 to step*t*.

6 $$ {P}_k^t=\rho {WP}_k^{t-1}+\left(1-\rho \right)M\left(:,k\right). $$

Meanwhile, if we take into account the labels for all proteins {p_1_, *p*_2_, ⋯, *p*_*m*_}, the above formula will be represented in matrix form as follows:

7 $$ {P}^t=\rho {WP}^{t-1}+\left(1-\rho \right)M. $$

According to the above formula, we can calculate the prediction matrix for the lncRNA-protein bipartite network.

## Results

### Datasets

At present, several commonly used public databases for lncRNA–protein interaction prediction include NPInter [21], NONCODE [22] and SUPERFAMILY [23].

In order to compare the prediction results with the prediction method proposed in reference [24], we used the same data set in the reference. For a detailed introduction to the data set, please refer to the literature [24]. The analyzed datasets were downloaded from: https://github.com/BioMedicalBigDataMiningLabWhu/lncRNA-protein-interaction-prediction.

### Evaluation metrics

In this section, we used a five-fold cross-validation method to assess the predictive performance of our proposed method. The test set was randomly divided into five subsets. Each time we run, one of the subsets was selected as the test set and the remaining four subsets were used as the training set. Afterwards, the training model was used to predict the test set and evaluate the performance of the model. To ensure that each subset would be tested, the process was repeated five times. Because there are some data deviations for each test, we have performed 20 times of five-fold cross-validation during the experiment and then average them as the final evaluation result.

We use seven evaluation metrics as follows: the area under the precision-recall curve (AUPR), the area under the receiver-operating characteristic curve (AUC), sensitivity, specificity, precision, accuracy, and F1-score.

8 $$ accuracy=\left( TP+ TN\right)/\left( TP+ TN+ FP+ FN\right), $$

9 $$ \mathrm{precision}=\mathrm{TP}/\left(\mathrm{TP}+\mathrm{FP}\right), $$

10 $$ \mathrm{recall}=\mathrm{TP}/\left(\mathrm{TP}+\mathrm{FN}\right), $$

11 $$ {\mathrm{F}}_1=2\times \left( precision\times recall\right)/\left( precision+ recall\right), $$

where TP denotes the number of true positives, *TN* denotes the number of true negatives, *FP* denotes the number of false positives, and *FN* denotes the number of false negatives.

All the evaluation indicators we mentioned above show that the larger the value, the better the performance.

### Performances

In this section, we compared the predictive ability of ACCBN with RWR [25], ProCF [26], LPIHN [13], and LPBNI [14].

**Fig. 3:**
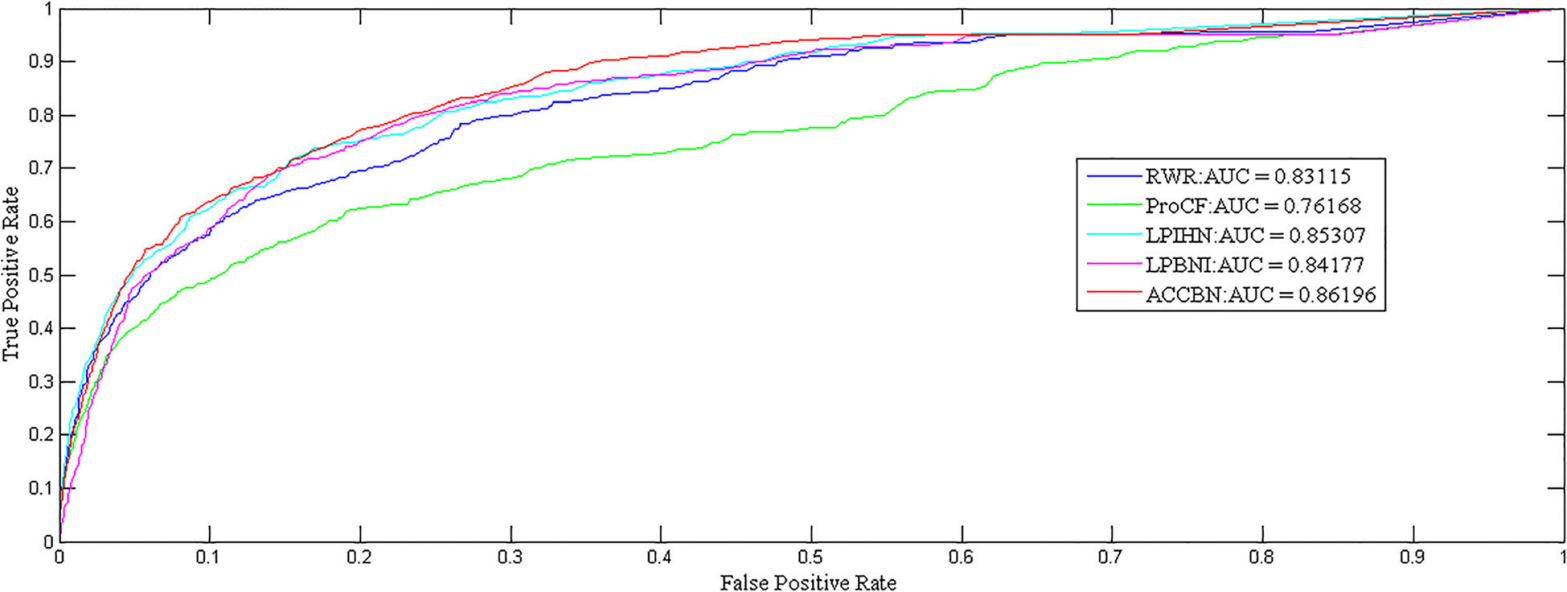
Performance comparison of different methods using ROC curves in predicting lncRNA–protein interactions Shown in the plot is the ROC for the whole dataset using RWR (blue, AUC: 0.83115), and ProCF (green, AUC: 0.76168),LPIHN (blue-green, AUC: 0.85307),LPBNI (purple, AUC: 0.84177), ACCBN(red, AUC: 0.86196), respectively

We performed a five-fold cross-validation experiment on the test dataset with the predictive models mentioned above to compare the results. The results are shown in Fig. 3 and Table 1. In Fig. 3, the ROC curve and the area under the curve (AUC) gained by various methods are shown. Obviously, the ACCBN method shows the best results. Furthermore, the AUC value obtained by the ACCBN method was 0.86196, which was significantly higher than the value of AUC obtained by using the RWR (0.83115), ProCF (0.76168), LPIHN (0.85307) and LPBNI (0.84177) methods respectively. The above results show that the ACCBN method has better predictive power than the RWR, ProCF, LPIHN and LPBNI methods. In order to verify the reliability of the ACCBN method, we compared the sensitivity, precision, accuracy, and F1-score of RWR, ProCF, LPIHN, LPBNI, and the ACCBN method respectively. As shown in Table 1, the ACCBN method exhibits a higher property in terms of sensitivity, precision, accuracy, and F1-score, compared with RWR, ProCF, LPIHN, and LPBNI methods.

**Table 1:** The property of different prediction methods

	sensitivity	precision	accuracy	F1-score
RWR	0.367965	0.353787	0.953597	0.360343
ProCF	0.29774	0.302855	0.950555	0.299193
LPIHN	0.371331	0.413918	0.958109	0.386821
LPBNI	0.4026	0.289802	0.943103	0.333666
ACCBN	0.460825	0.303115	0.962496	0.393211

To sum up, the ACCBN method can produce better predict results than the RWR, ProCF, LPIHN, and LPBNI methods in predicting unknown lncRNA-protein interactions.

## Discussions

There have been many studies on protein interaction at home and abroad [27]. There are also many websites which have unveiled a large protein response Database, such as STRING, GEN, BioGRID, DDBJ, Database of Interacting Proteins, ExPasy, Gepasi, etc. [28]. According to the relevant literature, the current studies on protein interaction data are broadly divided into the following three categories:

The first is to determine how proteins interact experimentally. For example, some of the websites mentioned above, such as DIP [29], record the protein data obtained by pure experiments, while other databases [28] also contain the data obtained through experiments. The characteristics of such research results are: the results are true and complete, and the items are complete and functional, but it takes a lot of time, and the preparation of experiments is complicated. However, you get a small amount of data finally. It is impossible to carry out a large number of experiments blindly.

The second is to predict the existence and function of protein interactions with biological theories. This kind of research relies on bioinformatics [27, 30]. Compared with the direct experiments, this kind of method USES some existing data to make predictions. But because there are so many types of protein, there may be a combination of quantity which is very large, the processing efficiency and can deal with the amount of data is still very limited.

The third category is computer algorithms that predict protein interactions. On the basis of the second method, in order to be able to process large data, there are many algorithms for computer prediction interaction [31–36]. This method is characterized by large-scale and high efficiency, which can provide more possibilities for the experiments, but since it is a prediction, there will be wrong results. Therefore, three important indicators to test the quality of such methods are computational accuracy, computational efficiency and how much data processed. Because of these advantages of computer methods, more and more researchers are seeking to use better algorithms to predict protein interactions.

The protein interaction network is huge and complex, and the protein reaction confirmed by experiments is only a small part at present. How to expand the known protein interaction network has become a major focus of the research on protein interaction. Biological experiments are time-consuming and expensive, and it is not feasible to test protein pairs one by one. So an effective method commonly used in bioinformatics to expand known protein interaction network rapidly is as follows: first forecast the potential of protein interactions with the known data and then predict the results of the experiment and verify them again.

Our article aimed at developing an efficient and accurate protein prediction method. Only by using the bipartite network prediction algorithm to predict protein interaction, there will be a lot of irrelevant data to reduce the coupling between the data and affect the prediction quality. The ACCBN uses ant colony algorithm to first conduct data clustering, and then constructs a bipartite network for prediction, solving the above problems effectively.

The above experimental results have shown that the prediction results of ACCBN are better than those of other comparison algorithms, and that the prediction results of ACCBN are better than those of other comparison algorithms as well.

**Fig. 4:**
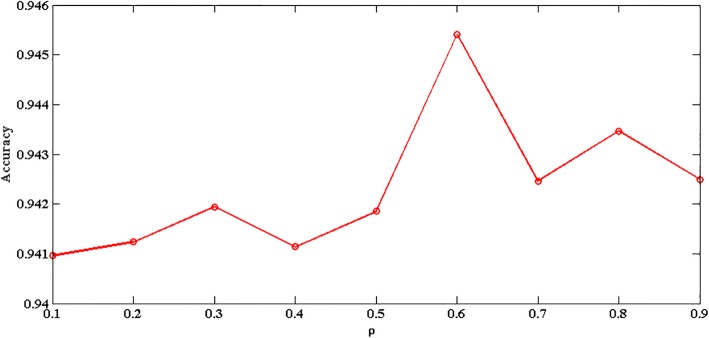
The relationship between parameters *ρ* and prediction accuracy

Let’s discuss the effect of parameters *ρ*on the prediction accuracy rate results, as shown in Fig. 4.

As can be seen from Fig. 4, as the *ρ* value increases, the value of the prediction accuracy also increases, but after*ρ* reaches 0.6, as the *ρ* value increases, the value of the prediction accuracy begins to decrease. The best prediction accuracy is obtained at*ρ* = 0.6. So we usually set *ρ* = 0.6 in the experiment.

## Conclusion

We proposed a novel prediction method for lncRNAs and proteins based on the known lncRNA-protein association bipartite and linear neighborhood similarity. We use the Ant-Colony-Clustering-Based Bipartite Network method (ACCBN) to predict unobserved lncRNA-protein associations. The experimental results show that the ACCBN method is superior to other comparison methods in predicting protein interactions. What’s more, the ACCBN method provides a new idea for researchers to identify key proteins by combining protein interaction information with other biological information.

## Abbreviations

ACCBN: Ant-Colony-Clustering-Based Bipartite Network method
AUC: Area under the curve
AUPR: Area under the precision-recall curve
FN: False negative
FP: False positive
LPBNI: lncRNA–protein bipartite network inference
LPIHN: lncRNA–protein heterogeneous network
ProCF: Protein-based collaborative filtering
RWR: Random walk
TN: True negative
TP: True positive
